# Drug-induced regeneration of pancreatic beta cells: An approach to cellular therapeutic targets

**DOI:** 10.1186/s13619-025-00255-9

**Published:** 2025-09-06

**Authors:** Parinaz Parsi, Saber Saharkhiz, Marzieh Ramezani Farani, Salar Bakhtiyari, Iraj Alipourfard

**Affiliations:** 1 Bioinformatics Lab, Department of Biology, School of Sciences, Razi University, Kermanshah, Iran; 2 Division of Neuroscience, Department of Cellular and Molecular Medicine, Faculty of Medicine, University of Ottawa, Ottawa, ON Canada; 3 Department of Biological Sciences and Bioengineering, NanoBio High-Tech Materials Research Center, Inha University, Incheon, Republic of Korea; 4 Department of Molecular Pharmacology and Physiology, Morsani College of Medicine, University of South Florida, Tampa, FL USA; 5 Institute of Physical Chemistry, Polish Academy of Sciences, Warsaw, Poland; 6 Department of Pharmaceutical Care and Pharmacotherapy, Faculty of Pharmacy, Medical University of Warsaw, Warsaw, Poland

**Keywords:** Beta cells, Regeneration therapy, Diabetes mellitus, DYRK1A, Transdifferentiation, Stem cells, Clinical translation

## Abstract

Diabetes mellitus is a common and serious metabolic disease globally, characterized by increased blood glucose levels. The major pathogenesis is the functional impairment of insulin-producing beta cells in the pancreas and the lack of insulin secretion. Although both type 1 and type 2 diabetes develop through distinct pathological mechanisms, they lead to the destruction and/or dysfunction of beta cells, resulting in inadequate beta cell mass to maintain normal blood glucose levels. For this reason, therapeutic agents capable of inducing beta cell proliferation can be considered a possible approach to restore beta cell abundance and treat type 1 and type 2 diabetes. Although several methods have been found to promote the replication of beta cells in animal models or cell lines, it is still challenging to promote the effective proliferation of beta cells in humans. This review highlights the different agents and mechanisms that facilitate pancreatic beta cell regeneration. Numerous small molecules have been discovered to influence beta cell proliferation, primarily by targeting cellular pathways such as DYRK1A, adenosine kinase, SIK, and glucokinase. Additionally, receptors for TGF-β, EGF, insulin, glucagon, GLP-1, SGLT2 inhibitors, and prolactin play critical roles in this process. Stem cell-based clinical trials are also underway to assess the safety and efficacy of stem cell therapies for patients with type 1 and type 2 diabetes. We have emphasized alternative therapeutic pathways and related strategies that may be employed to promote the regeneration of pancreatic beta cells. The knowledge raised within this review may help to understand the potential drug-inducible targets for beta cell regeneration and pave the way for further investigations.

**Graphical Abstract:**

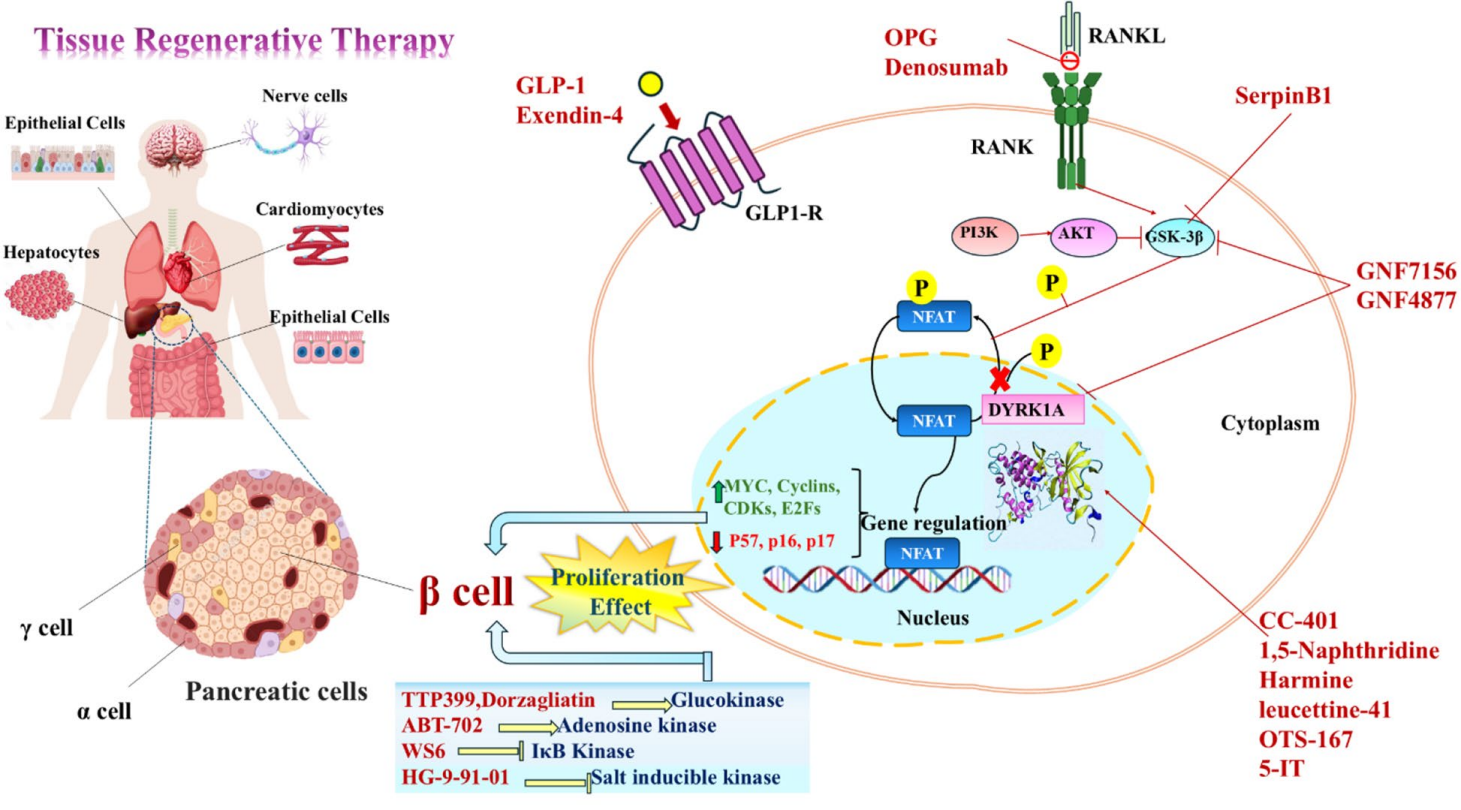

## Background

Numerous people throughout the world are living with diabetes mellitus (DM), a chronic metabolic disorder defined by consistently high blood glucose levels (Tan et al. 2019). Despite the advancements made in the management of diabetes, the prevalence of this condition continues to rise, necessitating the need for new and effective therapeutic approaches (Aloke et al. 2022). DM comprises two principal forms: type 1 diabetes (T1D), distinguished by an autoimmune degradation of pancreatic beta cells, and type 2 diabetes (T2D), predominantly linked to insulin resistance and decreased insulin secretion. In both forms, the decrease in inefficient beta cell mass plays a vital role, leading to insufficient insulin synthesis and ensuing hyperglycemia (Ojo et al. 2023). Beta cells, which are in the pancreatic islets of Langerhans, have the duty of generating and releasing insulin when blood glucose levels rise. The maintenance of a functional beta cell population is crucial for attaining sustained glycemic regulation (Ji et al. 2022). Hence, stimulating beta cell proliferation provides a compelling therapeutic approach for restoring insulin synthesis and potentially reversing the pathogenesis of diabetes (Pucelik et al. 2021).

The current therapeutic interventions for diabetes primarily revolve around the exogenous administration of insulin, oral antidiabetic medications, modifications to one's lifestyle, and, recently, microbiota interventions (Alipourfard et al. 2020; Docherty and Sussel 2021). Even though these methods assist in the management of blood glucose levels, they often need to be revised to achieve consistent glycemic regulation, hindering the progress of the disease and confronting the inherent beta cell deficiency (Ji et al. 2022). Consequently, innovative therapeutic methods that specifically aim to stimulate beta cell proliferation possess considerable potential in transforming the treatment of diabetes (Satin et al. 2021).

In both TD1 and TD2, there is a decline in the functionality of beta cells, resulting in their gradual loss. To improve hormone secretion, it is crucial to comprehend the cellular mechanisms (s) that govern beta cell mass (Docherty and Sussel 2021). Moreover, it is vital to determine the mitogenic factors that potentially promote beta cell proliferation in order to develop a therapeutic strategy that improves the prognosis of DM (Eguchi et al. 2022). The strategies to boost endogenous b-cell mass include (i) promoting the replication of beta cells, (ii) preventing the death of beta cells, (iii) inducing the generation of new beta cells, and (iv) regulating the transdifferentiation of beta cells (Shirakawa 2023). Efforts to promote beta cell proliferation using pharmaceuticals were initially met with significant challenges; however, chemicals that improve human beta cell proliferation in vitro were found through the use of high-throughput chemical screening (Wagner 2022). A range of small molecules has been found to modify the proliferation of beta cells. The drug agents induce proliferation mainly through various cellular targets, such as dual specificity tyrosine phosphorylation-regulated kinase 1A (DYRK1A), adenosine kinase, salt-inducible kinase (SIK), and glucokinase. Additionally, receptors for transforming growth factor- β (TGF-β), Endothelial growth factor (EGF), insulin, glucagon, glucagon-like peptide-1 (GLP1), and prolactin. Besides, the depletion of cell cycle regulators through oligonucleotide-mediated means has also been identified as an influential factor for beta cell proliferation (Goode et al. 2022). Knowledge about susceptible cellular targets of drug-induced proliferation can shed light on the identification of suitable agents and the optimal regeneration of pancreatic beta cells.

## Cellular targets for drug-induced cell regeneration

Regenerative medicine can address a wide range of medical conditions and tissue damage. Using small molecules and drugs in pharmacological approaches has become an important method for promoting cell regeneration (Esdaille et al. 2021). Recent preclinical and clinical findings suggest potential regenerative therapy in various organ systems and conditions such as dermal wounds, cardiovascular diseases, trauma, certain cancers, and more (Khan et al. 2020). Regenerative medicine involves using materials, newly generated cells, or combinations of both to replace missing tissue and contribute to tissue healing. Tissue regeneration holds tremendous potential across various medical disciplines, including orthopedics, dermatology, cardiology, and neurology, among others (Muruganandan and Wigerius 2020). The development of novel agents has played a pivotal role in enhancing regenerative therapies' efficacy and success rates. These agents encompass a diverse range of techniques, including stem cell therapy, growth factors, biomaterials, gene therapy, and tissue engineering (Mao and Mooney 2015). A revolution started in regenerative medicine with the identification of induced pluripotent stem cells. It led to the advancement of direct reprogramming, the process of changing the cell type without returning to a stem cell state. This process has successfully produced various cell types from fibroblasts, such as neurons, cardiomyocytes, endothelial cells, hematopoietic stem/progenitor cells, and hepatocytes (Sadahiro et al. 2015). The following are some potential cell types and their drug-targeted stimulation to proliferate that hold promise for regenerative medicine applications (Table 1).

**Table 1:** Pharmacological agents have been trialed for cell regeneration in different cells

Cell type	Drug agent	Regulation	Mechanism	Outcome	Ref
Cardiomyocytes	Carbacyclin	up	A PPARά agonist and inducible overexpression of constitutively active PPARδ in cardiomyocytes	Enhanced cardiac function	Magadum et al. 2017
	Pkm2	up	Delivery of Pkm2 modRNA	Boosted heart cell division	Magadum et al. 2020
	CDK1, CDK4, Cyclin B1 and Cyclin D1	up	Delivery of recombinant CDK1, CDK4, Cyclin B1 and Cyclin D1	Stimulated division of heart muscle cells	Mohamed et al. 2018
Hepatocytes	bardoxolone methyl (CDDO-Me)	up	activating Nrf2	Improved liver regrowth and function	Chan et al. 2021
	Mfap4	up	knockdown Mfap4 by siP70S6k	Promoted cell growth speeds up liver regeneration and reduces fibrosis	Iakovleva et al. 2023
Epithelial cells	UC-MSCs and clinical-grade MSCs	up	Delivery of UC-MSCs or clinical-grade MSCs (mesenchymal stem cells)	Lowered inflammatory markers and supported lung recovery	Hashemian et al. 2021; Liang et al. 2020
	MenSCs	up/down	Delivery of MenSCs to lung tissue	Improved cell survival	Xiang et al. 2017
	Paeoniflorin	up	Regulating intestinal stem cells (ISCs)	Promoted colon organoid growth, increased regeneration, and differentiation markers	Ma et al. 2023

### Cardiomyocytes

Cardiomyocytes are the specialized cells responsible for the contraction of the heart. In regenerative medicine, the ability to replace damaged or lost cardiomyocytes is crucial for treating heart diseases such as myocardial infarction (Wang et al. 2023). Different approaches, including the use of stem cells or direct reprogramming of other cell types into cardiomyocytes, are being explored to regenerate heart tissue. Animal studies have shown that adult mammalian heart cells can be boosted to renew themselves through interventions like regulating transcription factors, stimulating specific microRNA pathways, exposing them to low oxygen conditions, or activating certain growth factor signaling pathways (Garbern and Lee 2022). Transcription factors that play a role in the initial differentiation of cardiomyocytes also support the proliferation of cardiomyocytes. These factors include GATA4, NF-κβ, TBX20, CSL activated by Notch, and TCF/LEF activated by Wnt/β-catenin (Wang et al. 2023). Some mechanisms, such as the activation of transcription factors like GATA4 and NF-κβ, as well as pathways like Wnt/β-catenin, are utilized by pancreatic beta cells during regeneration. For instance, pancreatic beta cells depend on factors such as NF-κβ and Wnt/β-catenin to enhance cellular proliferation and differentiation (Kroon et al. 2008). Carbacyclin was shown to be a tiny molecule that stimulates the proliferation of cardiomyocytes in one study that used a Fucci-based approach to screen chemical compound libraries. Carbacyclin was found to induce the proliferation of neonatal and adult mononuclear rat cardiomyocytes through a δ (PPARδ)/PDK1/p308Akt/GSK3β/β-catenin pathway in vitro (Magadum et al. 2017). Another research study showed that administering Pkm2-modified RNA (modRNA) to the hearts of mice has been shown to enhance the proliferation of cardiomyocyte cells and cardiac function following myocardial infarction (Magadum et al. 2020). Moreover, a mixture of four cell-cycle regulators, which make up the CDK1: CCNB and CDK4:CCND complexes, can effectively stimulate the proliferation of cardiomyocytes and promote their survival both in vivo and in vitro (Mohamed et al. 2018).

### Hepatocytes

Hepatocytes are the main functional cells of the liver and are involved in various essential metabolic functions. Liver diseases and injuries can significantly impact liver function. Utilizing hepatocytes or their progenitor cells for regenerative purposes may support liver repair and restore normal liver function (Kholodenko and Yarygin 2017). Key elements in signaling pathways are crucial for enhancing liver regeneration and promoting hepatocyte proliferation and differentiation. Understanding these elements is important for developing therapeutic inducers to improve liver regeneration in tissue engineering and clinical regenerative therapy (Hora and Wuestefeld 2023). One study suggested that activating Nrf2 with the stimulator compound bardoxolone methyl (CDDO-Me) is a promising approach to improve liver regeneration. This potent Nrf2 activator enhances liver volume restoration and function in an Nrf2-dependent manner by promoting hepatocyte growth and proliferation, suppressing immune and inflammatory signals, and inducing metabolic changes in the remaining liver tissue (Chan et al. 2021). Also, activating Nrf2 specifically in β-cells enhances their proliferation and mass, leading to improved glucose tolerance. When human islets are transplanted under the kidney capsule of immunocompromised mice and treated with bardoxolone methyl, a systemic Nrf2 activator, there is an increase in β-cell proliferation. By managing reactive oxygen species levels, Nrf2 regulates β-cell mass, making it a promising therapeutic target for expanding and safeguarding β-cell mass in diabetes treatment (Baumel-Alterzon et al. 2022). Additionally, a research study performed a genetic screen in living organisms and discovered that reducing the expression of microfibril-associated protein 4 (Mfap4) in liver cells promotes cell growth, accelerates liver regeneration, and decreases liver fibrosis. The targeting of Mfap4 also influences mTOR and other pathways involved in liver regeneration. This study indicates the possibility of using siRNA-based therapies to enhance liver regeneration (Iakovleva et al. 2023).

### Epithelial Cells

Epithelial cells are the building blocks of many tissues and organs, such as the skin, lungs, and intestines. They are essential for preserving the integrity of tissues and acting as a barrier. Regenerative approaches targeting epithelial cells aim to restore damaged epithelial tissues, promote wound healing, and improve organ function (Karathanasis 2014). For example, the function of lung epithelial cells in repairing and regenerating the lung depends on crucial signals from the surrounding environment (Khedoe et al. 2021). In some trials, the administration of UC-MSCs (umbilical cord-derived mesenchymal stem cells) or clinical-grade MSCs (mesenchymal stem cells) may have potential benefits in the regeneration of epithelial cells. These benefits include the reduction of inflammatory cytokines such as TNF-α, IFN-γ, IL6, IL8, and C-reactive protein. The treatment with UC-MSCs or clinical-grade MSCs may also contribute to promoting lung recovery in patients who have survived. This suggests that these types of stem cell therapy could potentially be valuable in addressing inflammation and aiding in the recovery of lung function in patients (Hashemian et al. 2021; Liang et al. 2020). However, it's important to note that further research and larger clinical trials would be needed to confirm and expand upon these findings in the case of epithelial cells of different tissues. Menstrual blood-derived stem cells (MenSCs) from menstrual blood can treat diseases like acute lung injury (ALI) by migrating to the lung, reducing inflammation, and promoting lung tissue repair. Because of this, MSCs may represent a novel approach to treat LPS-induced ALI, and they may even have therapeutic promise for ALI in general (Karimi et al. 2024; Xiang et al. 2017). MenSCs were found in the lungs using live imaging. They were found to improve pulmonary microvascular permeability, reduce histopathological damage, downregulate IL-1, and upregulate IL-10 in bronchoalveolar lavage fluid and the damaged lung. Immunohistochemistry revealed that MenSCs had a positive impact by increasing PCNA expression and decreasing caspase 3 expression. Keratinocyte growth factor (KGF) is an essential element of epithelial cells, which was also upregulated after MenSCs administration in lung epithelial cells. In vitro studies showed that MenSCs can increase the viability of BEAS-2B cells and impede LPS-induced apoptosis as an inflammatory pathogenic process of lung injury. These results point to the possibility of MenSC-based treatments for ALI and the regeneration of lung epithelial cells as possible future treatments (Xiang et al. 2017). Yujing Ma et al. studied the effects of Paeoniflorin (PF) on intestinal stem cells (ISCs) to enhance intestinal epithelium regeneration and repair in ulcerative colitis (UC). Their findings demonstrated that PF reduced colitis induced by DSS and improved intestinal mucosal injury by regulating ISCs through the PI3K-AKT-mTOR signaling pathway. In vitro, PF enhanced the development of TNF-α-induced colon organoids and upregulated genes and proteins associated with intestinal stem cell (ISC) differentiation and regeneration. Additionally, PF improved the ability of LPS-induced IEC-6 cells to repair themselves (Ma et al. 2023). Bioactive compounds such as Paeoniflorin (PF), which influence the PI3K-AKT-mTOR signaling pathway in intestinal epithelial cells, may also be utilized to promote beta cell repair. This is because the PI3K-AKT-mTOR pathway is crucial for controlling cell survival and proliferation in beta cells.

## Transdifferentiation of non-beta cells into beta cell

Cellular transdifferentiation, or lineage reprogramming, facilitates beta-cell regeneration. Hepatic, gastrointestinal, and pancreatic exocrine cells share common endodermal progenitors, enabling efficient transdifferentiation into beta cells. Due to similar developmental transcription mechanisms and epigenetic landscapes, minimal rearrangement of the epigenome is required, making this process appealing for cellular reprogramming (Wang and Zhang 2021). Targeted modulation of transcription factors is essential for pancreatic cell differentiation and function, facilitating the conversion of non-beta cells into functional, glucose-responsive beta cells (Spezani et al. 2024). One of the major strategies includes ectopic expression of gene regulators, such as PDX1, PAX4, NGN3, and MAFA, to induce the reprogramming of pancreatic α-, δ-, and exocrine cells, along with non-pancreatic cells like hepatocytes, gallbladder, and gastrointestinal cells, into beta-like cells (Ber et al. 2003; Baeyens and Bouwens 2008; Zhang et al. 2016; Meivar-Levy and Ferber 2015; Baeyens et al. 2005). In 2003, Sara Farber and colleagues found that in the process of differentiating primary gut endodermal and mature hepatic cell lines into pancreatic cells, PDX-1 is an essential factor. The conversion of the liver to the pancreas may provide a novel approach for generating endocrine-pancreatic tissue to replace dysfunctional beta cells in individuals with diabetes (Ber et al. 2003). Also, in 2021, Sara et al. used PDX1, NEUROD1, and MAFA to produce IPC from liver cells. PDX1 triggered mesoderm-to-endoderm trans-differentiation, NEUROD1 amplified PDX1's effects by binding to the same location, and two days after differentiation began, MAFA was activated to boost insulin production by maturing IPC (Lee et al. 2021). In addition, by directly supplementing three pancreatic transcription factors—PDX1, PAX4, and MAFA—in a hierarchical order, Ferber et al. demonstrated that mature beta cell-like traits may be generated (Berneman-Zeitouni et al. 2014). Baeyens and colleagues transduced adult human pancreatic exocrine cells with lentiviruses expressing activated MAPK and STAT3 and cultured them as monolayers or 3D structures. Expression of STAT3 and MAPK-activated neurogenin 3 in 50%~80% of the transduced exocrine cells. Only exocrine cells cultivated in suspension prior to 3D culture showed a rise in insulin-positive cells in this experiment (Lemper et al. 2014).

Alpha cells have emerged as a promising approach to replace the lost beta cell mass, which could potentially lead to a cure for diabetes. This method is designed to recover insulin production in patients with diabetes by transforming the hormone-secreting alpha cells, which are typically associated with glucagon production, into insulin-producing beta cells (Saleh et al. 2021). For instance, Cheng-Ho Chung et al. presented a model in 2010 for beta-cell neogenesis in which mature alpha-cells rapidly replicate and convert efficiently into beta cells following PDL and alloxan treatment. This could provide an attractive model for studying the mechanism of alpha-cell to beta-cell conversion (Chung et al. 2010).

Regarding the differentiation of beta cells, researchers found that an exocrine enzyme released during pancreatitis, trypsin, and its receptor, protease-activated receptor 2 (PAR2), are of importance. High levels of PAR2 have been detected in the islets, including beta cells, and experiments with a PAR2 agonist and knockout mice confirmed its necessity for alpha-to-beta cell trans differentiation (Piran et al. 2016). Additionally, PAR2 was implicated in broader regenerative processes, such as liver regeneration. The destruction process of beta cells is related to the suppression of PAR2 activation. Importantly, glucagon-insulin double-positive cells, which are intermediates in transdifferentiation, could be induced using a PAR2 agonist alongside an insulin secretion inhibitor (Winzell and Levine 2022).

## Stem cell contributions to beta-cell regeneration

Stem cells offer a promising approach for treating diabetes and its complications due to their ability to modulate the immune system, differentiate into multiple cell types, and regenerate damaged tissues. Embryonic, induced pluripotent, and adult stem cells, such as umbilical cord blood stem cells (UCB), peripheral blood mononuclear cells (PB-MNCs), and bone marrow-derived cells like bone marrow mesenchymal stromal cells (BMMSCs) and hemopoietic stem cells (BM-HSCs), are among the numerous stem cell types that have been investigated for their capacity to generate new insulin-producing cells (Peng et al. 2018; Hamad et al. 2021; Soria et al. 2008). A study conducted by Douglas A. Melton and colleagues, using a scalable differentiation technique, was able to generate hundreds of millions of beta cells that respond to glucose in vitro from human pluripotent stem cells (hPSCs). In response to glucose, these beta cells derived from stem cells (SC-b) release Ca^2^⁺, store insulin in secretory granules, and release levels of insulin that are similar to adult beta cells when tested with different glucose challenges. Moreover, these cells significantly alleviate hyperglycemia in diabetic mice after transplantation and produce human insulin into the circulation in a glucose-regulated pattern (Pagliuca et al. 2014). Additionally, research on pancreatic and hepatic stem cells has also been conducted, notably in a pioneering study by Kevin A. D’Amour and colleagues in 2006 and 2008. They developed a method to differentiate human embryonic stem (hES) cells into endocrine cells that produce key pancreatic hormones, including insulin, glucagon, somatostatin, pancreatic polypeptide, and ghrelin. This approach resembles natural pancreatic development by guiding cells through stages like definitive endoderm, gut-tube endoderm, pancreatic endoderm, and endocrine precursors. The achieved insulin-producing cells have insulin levels comparable to those in adult islets and release C-peptide in response to various stimuli, although their response to glucose is minimal (D’Amour et al. 2006; Kroon et al. 2008).

A growing number of stem cell-based clinical trials are currently underway to evaluate the safety and efficacy of stem cell therapies in patients with TD1 and TD2. These trials include both autologous and allogeneic stem cell approaches, with a variety of cell types under investigation, including hematopoietic stem cells, mesenchymal stem cells (MSCs), and stem cell-derived pancreatic progenitors or beta cells (Sali et al. 2025). Clinical trials using MSCs have reported improved metabolic control, reduced insulin requirements, and modulation of the immune response in TD1 and TD2 patients. These effects are likely mediated by the immunomodulatory and anti-inflammatory properties of MSCs rather than their differentiation into beta cells (Hering et al. 2025; Lu et al. 2021). Recent clinical trials have investigated innovative stem cell-based therapies for type 1 diabetes mellitus (T1DM). A pilot study (NCT03920397) combined intravenous administration of allogeneic mesenchymal stem cells (MSCs) with oral vitamin D, reporting stable increases in basal C-peptide levels over six months (Dantas et al. 2021). Another Phase I/II trial (NCT04078308) involving newly diagnosed TD1M patients revealed that MSC infusion improved HbA1c levels, increased C-peptide levels, and shifted cytokine profiles from pro-inflammatory to anti-inflammatory. Early-stage MSC transplantation showed greater efficacy compared to late-stage administration. However, the small sample sizes in many MSC studies limit the reliability of clinical conclusions (Izadi et al. 2022). Human pluripotent stem cells (hPSCs), including human embryonic stem cells (hESCs) and induced pluripotent stem cells (hiPSCs), also present a promising therapeutic approach. Current trials are focused on transplanting either terminally differentiated β-like cells or pancreatic progenitor cells co-expressing PDX1 and NKX6.1, which are critical markers for β-cell lineage commitment (Bruin et al. 2014; Rezania et al. 2013). ViaCyte’s initial trial (NCT02239354) tested hESC-derived progenitor cells (PEC-01) encapsulated in an immunoprotective device (PEC-Encap). However, insufficient vascularization led to graft failure and no insulin production (Henry et al. 2018; Ramzy et al. 2021). A subsequent trial (NCT03163511) utilized an improved vascular-permeable device (PEC-Direct), resulting in successful engraftment in 63% of patients and detectable C-peptide secretion in 35%, without severe rejection events. Further optimization, including higher cell doses and enhanced device perforation, improved C-peptide levels and reduced insulin dependence in selected patients (Shapiro et al. 2021).

To address immune rejection, ViaCyte and CRISPR Therapeutics developed VCTX210A—CRISPR-edited progenitor cells that lack HLA class I and express PD-L1, encased in a removable, vascularized device (NCT05210530; ongoing). Additionally, a modified PEC-Encap device developed with Gore materials aimed to enhance vascularization without requiring immunosuppression (NCT04678557); results are pending (Ramzy et al. 2023).

## Inducing beta cell proliferation through therapeutic targeting

**Fig. 1:**
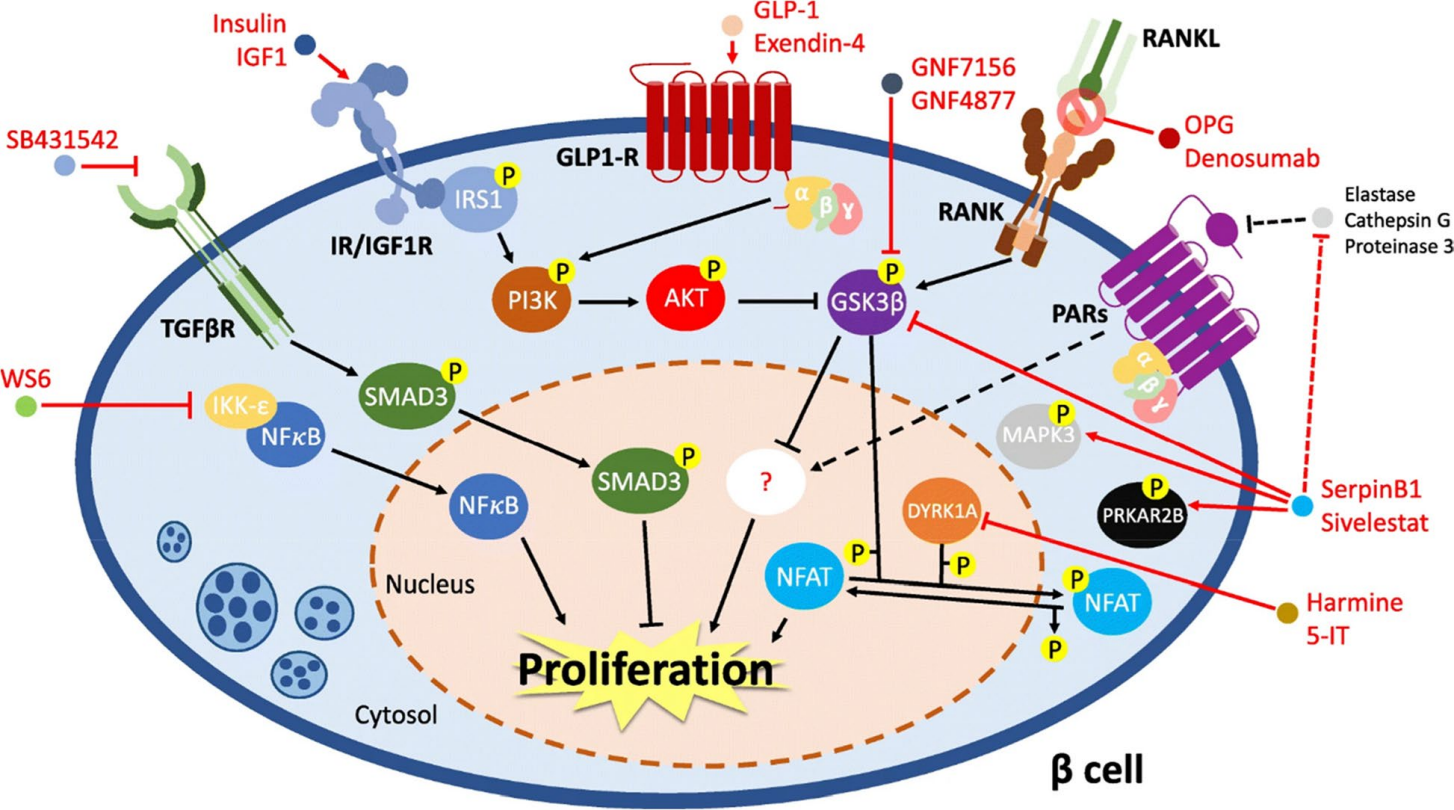
Mechanisms at the molecular level that control the proliferation of human beta cells. The molecular mechanisms regulating human beta cell proliferation are complex and involve a combination of intrinsic and extrinsic factors. NFκB is normally restrained in the cytosol by IKK-ε. WS6 blocks this inhibition, allowing NFκB to enter the nucleus and promote cell growth. Through the activation of SMAD3, the TGF-β pathway influences the proliferation of beta cells. Inhibiting SMAD3 activation is how SB431542, a TGF-βR inhibitor, stimulates cell proliferation. Beta cell regeneration is initiated by signaling pathways that include insulin receptors (IR), insulin-like growth factor 1 receptor (IGF1R), and glucagon-like peptide 1 receptor (GLP-1R). These pathways modulate the PI3K-AKT axis and decrease the activity of GSK3β. GNF7156, GNF4877, GSK3β inhibitors, osteoprotegerin (OPG), and denosumab also inactivate GSK3β. OPG and denosumab prevent the interaction of RANKL with RANK, preventing activation of the extrinsic apoptotic pathways. After likely inhibiting proteases such as elastase, cathepsin G, or proteinase 3, SerpinB1 and sivelestat promote the proliferation of human beta cells by elevating the phosphorylation levels of MAPK3, PRKAR2B, and GSK3β, to stimulate mitogenic pathways in human beta cells, small compounds such as harmine or 5-iodotubercidin (5-IT) can block DYRK1A, which in turn reduces the NFAT phosphorylation state (adapted from Basile et al. 2019)

The exploration of pharmacological substances that protect beta cells and stimulate their proliferation has been critically studied in the field of diabetes cell therapy. These substances endeavor to maintain the present beta cell population, prevent beta cell death, and trigger beta cell replication (Table 2). This section presents several remarkable discoveries in this specific field: Major intracellular targets, their mechanistic roles, and effective therapeutic agents are demonstrated in Fig. 1.

**Table 2:** Potential tissue targets trailed by pharmacological agents to modulate the proliferation and regeneration of beta cells

**Target(s) or pathway(s)**	**Agent/chemical/factor**	**Agent family**	**Effects on tissue**	**Effect of agent at Target**	**Ref.**
PI3K/Akt and CREB-IRS2	Gaba	Endogenous small molecule	↑ beta cells proliferation	Synergistic activation/Inhibition	Hagan et al. 2022
GLP1R	Exendin 4-based agents: Exenatide and lixisenatide GLP-1-based agents: albiglutide, dulaglutide, liraglutide, and semaglutide Dual GIP/GLP-1 receptor agonists: Tirzepatide, NNC0090-2746	Synthetic peptide	↑ beta cells proliferation, beta cells protection	Synergistic activation	von Herrath et al. 2021; Fusco et al. 2017; Anderson and Trujillo 2016; Knop et al. 2023; Wajcberg and Amarah 2010; Bonora et al. 2021; Coskun et al. 2018
Prolactin receptor and growth hormone receptor (JAK-STAT signaling)	Placental lactogen; prolactin	Endogenous humoral	N/A	Inhibition	Fujinaka et al. 2007; Yamamoto et al. 2010
DYRK1A	CC-401, 1,5-Naphthridine, Harmine, leucettine-41, OTS-167, 5-Iodotubercidin(5-IT)	Small molecule	↑ beta cells proliferation	Inhibition	Bain et al. 2007; Naert et al. 2015; Allegretti et al. 2020; Dirice et al. 2016a; Abdolazimi et al. 2018
DYRK1A, GSK-3β	Aminopyrazine (GNF7156, GNF4877), 6-Azaindole derivative (GNF2133)	Small molecule	↑ beta cells proliferation	Inhibition	Liu et al. 2020a, 2020b
RANKL	Denosumab	Monoclonal antibody	↑ beta cells proliferation	Inhibition	Kondegowda et al. 2015
RANKL, GSK3	Osteoprotegerin	Endogenous humoral	↑ beta cells proliferation	Inhibition	McClung et al. 2006
Proteases	Serpin B1	Endogenous humoral	↑ beta cells proliferation	Inhibition	El Ouaamari et al. 2016
Glucokinase	Glucokinase activators (e.g., TTP399, Dorzagliatin)	Small molecule	↑ beta cells proliferation	Activation	Porat et al. 2011
Adenosine kinase	ABT-702	Small molecule	↑ beta cells proliferation	Activation	Annes et al. 2012a
Adenosine kinase/DYRK1A	5-Iodotubercidin (5-IT)	Small molecule	↑ beta cells proliferation	Inhibition	Annes et al. 2012a
Adenosine receptor (ADORA2A)	5-N-ethyl carboxamideadenosine (NECA)	Small molecule	↑ beta cells proliferation	Inhibition	Andersson et al. 2012
EGFR/NFkB pathways (IκB kinase and EBP1)	WS6	Small molecule	↑ beta cells proliferation	Activation	Shen et al. 2013
Salt-inducible kinases	HG-9–91-01	Small molecule	↑ beta cells proliferation (Concentration-dependent)	Inhibition	Huang et al. 2022; M. Kim, 2004

### GABA

The neurotransmitter gamma-aminobutyric acid (GABA) functions as a signaling molecule that is secreted from beta cells. Its importance in insulin secretion stimulation in human beta cells and insulin exocytosis has been well-documented (Hagan et al. 2022). Furthermore, dysregulation of GABA signaling has been observed in beta cells derived from patients with TD2 (Korol et al. 2018). In addition to its function in the regulation of insulin, GABA has the capacity to induce the growth of beta cells in rodent and human beta cells (Soltani et al. 2011). Through activating a calcium-dependent signaling pathway as well as the downstream PI3K/Akt and CREB-IRS2 signaling pathways, GABA triggers the replication of beta cells in transplanted human islets. The findings presented by Purwana and colleagues indicate that GABA induces the influx of calcium ions in human beta cells, resulting in the subsequent phosphorylation of AKT and cAMP response element binding protein (CREB). This intriguing finding suggests that GABA transmits its signals via the PI3K/AKT pathway. Furthermore, in a trial with GABA treatment, an enhanced IRS-2 mRNA expression was found. 

In vivo, GABA has been found to stimulate the conversion of α-to beta-like cells, according to a recent study of the Collombat group. This process mobilizes duct-lining precursor cells that initially take on an α-cell identity before transforming into functional beta-like cells through sustained GABA exposure. The in vivo reversal of chemically induced diabetes is achieved by these newly produced beta-like cells. It has been suggested that comparable conversion mechanisms occur in people when GABA is administered to transplanted human islets, leading to a reduction in α-cell counts and a surge in beta-like cell numbers. The GABA-induced beta-like cell is mediated by α-cells (Ben-Othman et al. 2017; Li et al. 2017).

### GLP-1 and Exendin-4

Glucose administration through the gastrointestinal tract can increase insulin secretion higher than administering it via peripheral circulation, a phenomenon known as the “incretin effect” (Holst 2019). This effect is mediated by the gut hormone GLP-1, which is released after food ingestion and stimulates beta cells to produce more cAMP and secrete more insulin (Doyle and Egan 2007). In the absence of high glucose, GLP-1 has little impact on insulin production, minimizing the chance of hypoglycemia. Basic researchers, medical professionals, and pharmaceutical companies have all taken an interest in the incretins. Because of this, new medications have been developed at a quick pace, mostly to treat TD2 (Kim and Egan 2008). These medications include dipeptidyl peptidase 4 (DPP4) degrading enzymes, GLP-1R agonists, and incretin hormone inhibitors. By focusing on DPP4, the half-lives of GLP-1 and gastric inhibitory polypeptide (GIP) are prolonged, resulting in increased secretin hormone levels in the blood (Satin et al. 2021; Holst 2019). A combination of GLP-1/GIP and glucagon/GLP-1 agonists are being evaluated in several studies. Dual GLP-1/GIP agonists have shown promising metabolic results (Rizvi and Rizzo 2022). Native GIP and GLP-1 are ineffective in treating type 2 diabetes due to their short half-lives. GLP-1RAs that have been authorized by the FDA are similar to exedin-4 or GLP-1 (Liu 2024). Albiglutide, dulaglutide, liraglutide, and semaglutide are GLP-1 based, whereas exenatide and lixisenatide are based on exendin-4. With significant co-agonism at both receptors, tirzepatide is only FDA-approved dual GIP/GLP-1 receptor agonist. Differences in pharmacokinetics between short- and long-acting analogs significantly affect their action, efficacy, and tolerability (Alfaris et al. 2024; Gallwitz 2022). The ongoing enhancement in the pharmacokinetic and pharmacodynamic characteristics of these drugs has led to their expansion of use beyond TD2 and obesity, including a decrease in cardiovascular events (Ciardullo et al. 2024).

Combining the GLP1R agonist, liraglutide, with an anti-interleukin (IL)−21 antibody was the alternative technique used in a clinical trial on people who had just been diagnosed with TD1. Stress-related beta cell death can be attenuated by GLP1R agonists, and IL-21 can be used to attract cytotoxic CD8 + T lymphocytes to the pancreatic islets. The decline in beta cell function associated with TD1 was considerably mitigated after one year of treatment when the anti-IL-21 antibody and liraglutide were used together (von Herrath et al. 2021).

#### Exendin 4-based agents

Exendin-4 is a GLP-1 analog that improves glucose tolerance in diabetic patients by enhancing insulin secretion. It mimics the action of human GLP-1, increasing glucose-dependent insulin secretion, impeding glucagon release, slowing gastric emptying, and promoting satiety (Ciardullo et al. 2024). Joseph Fusco et al. have shown that exendin-4 increased beta cell mass and proliferation by means of EGFR, which clarifies the involvement of EGFR signaling in exendin-4's impacts on beta cell mass and plasma glucose metabolism (Fusco et al. 2017a). Different exenatide formulations, such as the short-acting (Byetta, exenatide IR) and long-acting (Bydureon, exenatide ER) varieties, were developed after the identification of exendin-4. Both have proven effective in improving glycemic control, reducing body weight, and providing cardiovascular benefits. Byetta, a synthetic version of exendin-4 with enhanced stability, was the first FDA-approved GLP-1 receptor agonist for treating TD2 in 2005. The FDA approved Bydureon, the first once-weekly GLP-1 receptor agonist injection for adults with TD2, in 2012 (Liu 2024; Brunton and Davidson 2016).

Lixisenatide is a synthetic peptide derived from exendin-4 with a strong binding affinity for the GLP-1 receptor, about four times higher than GLP-1 itself. In the treatment of TD2, the once-daily dose is suggested despite its short half-life of 2~4 h. Nevertheless, lixisenatide is currently available in the United States alongside insulin glargine (iGlarLixi®); it is no longer sold alone as of 2023 (Anderson and Trujillo 2016; Ahrén et al. 2013).

#### GLP-1-based agents

The development of GLP-1 receptor agonists (GLP-1RAs) is centered around the creation of GLP-1 derivatives with modified properties that make them resistant to the action of the DPP-4 enzyme and have slow renal clearance. The goal of these modifications is to increase the GLP-1 receptor stimulation beyond what is normally physiologically possible and to prolong the pharmacological half-life of the GLP-1 hormone (Liu 2024). Common GLP-1-based agents include liraglutide, semaglutide, dulaglutide, and albiglutide. These medications have shown effectiveness in enhancing blood sugar regulation, promoting weight loss, and providing cardiovascular advantages, making them highly beneficial for individuals with diabetes and obesity (Knop et al. 2023); Bush et al. 2009; Neeland et al. 2021; Miles and Kerr 2018; Wajcberg and Amarah 2010; Bonora et al., 2021). Notably, liraglutide and semaglutide have achieved substantial decreases in HbA1c levels and body weight, with semaglutide additionally receiving approval for long-term weight management (Wajcberg and Amarah 2010; Knop et al. 2023). Albiglutide and dulaglutide are GLP-1 analogs that contain an alanine-to-glycine substitution at position 8, rendering these peptides resistant to DPP-4 enzymatic activity. Both medications were approved by the FDA in 2014 for the treatment of TD2M (Bush et al. 2009). Dulaglutide was also approved in 2020 to lower the risk of major adverse cardiovascular events (MACE) in persons with TD2 who already have cardiovascular disease or a number of related risk factors (Bonora et al. 2021).

#### Dual GIP/GLP-1 receptor agonists

When compared to GLP-1 receptor agonists alone, the unimolecular dual incretin receptor agonist offers a more natural approach to addressing manifestations related to TD2. Two dual GIP/GLP-1 receptor agonists have been identified in clinical trials. The initial dual agonist is NNC0090-2746, a 40-amino acid peptide that has a C16:0 fatty acid attached to lysine at position 40; it is also called RG7697, RO6811135, or MAR709 (Liu 2024). Tirzepatide, the second dual agonist, is distributed under the brand names Mounjaro® for TD2 and Zepbound® for obesity. It was formerly known as LY3298176. The unit is a 4.8 kDa peptide with 39 amino acids that is acylated with a C20:0 fatty acid at lysine position 20 (Coskun et al. 2018).

#### SGLT2 inhibitors

SGLT2 and SGLT1 are responsible for actively transporting glucose across the proximal convoluted tubule (PCT) cells in the kidney, each with different capacities. SGLT2, a high-capacity and low-affinity transporter, is primarily located in the S1 segment of the PCT (Saisho 2020). It is believed to be responsible for about 90% of glucose reabsorption, with its expression restricted to the kidney. In uncontrolled Type 2 Diabetes Mellitus (TD2M), elevated plasma glucose levels surpass the kidney's glucose reabsorption capacity, saturating the SGLT receptors and leading to increased glucose excretion in the urine (Morace et al. 2024). A preclinical study of cultured proximal convoluted tubule (PCT) cells from human patients has shown that individuals with TD2M have significantly higher expressions of SGLT2 and GLut2 compared to healthy individuals (Huang et al. 2023). Blocking SGLT to suppress glucose reabsorption can lead to increased glucose excretion in urine, thereby lowering plasma glucose levels and potentially providing a new treatment approach for TD2M without the side effects of current medications (Saisho 2020). However, a concern with this method is that it might negatively impact kidney function, causing excessive water loss through urine and leading to dehydration. SGLT2 inhibitors help manage hyperglycemia, decrease body weight and visceral fat, and enhance metabolic issues linked to metabolic syndrome, including blood pressure, lipid levels, and serum uric acid levels (Saisho 2020; Morace et al. 2024). In Japan, ipragliflozin was the first SGLT2 inhibitor introduced to the market in 2014. Currently, six SGLT2 inhibitors are available for treating type 2 diabetes: ipragliflozin, dapagliflozin, canagliflozin, empagliflozin, luseogliflozin, and tofogliflozin. Additionally, ipragliflozin and dapagliflozin have been approved for treating type 1 diabetes in Japan (Dixit et al. 2025; Abdul-Ghani et al. 2013). Recent cardiovascular outcome trials (CVOTs) have demonstrated that SGLT2 inhibitors improve cardiovascular and renal outcomes in patients, both with and without type 2 diabetes mellitus (TD2M). Consequently, the American Diabetes Association and the European Association for the Study of Diabetes (EASD) recommend SGLT2 inhibitors as a primary treatment for TD2M (Buse et al. 2020). Cheng et al. (2016) investigated the potential of the SGLT2 inhibitor empagliflozin to preserve pancreatic β-cell mass and improve glycemic control in a streptozotocin (STZ)-induced type 1 diabetes mouse model. Their findings demonstrated that empagliflozin treatment significantly enhanced glucose tolerance and elevated both serum insulin levels and insulin mRNA expression. Immunohistochemical analysis revealed an increase in β-cell area and proliferation (as indicated by Ki-67 co-staining with insulin), alongside reduced β-cell apoptosis and oxidative stress. These results suggest that empagliflozin may support β-cell regeneration and function in type 1 diabetes, potentially by mitigating glucotoxicity-induced oxidative damage (Cheng et al. 2016). Also, in 2022 study, Daniel Karlsson investigated the effects of dapagliflozin, an SGLT2 inhibitor, on human islets transplanted into diabetic mice. The treatment enhanced survival rates, reduced blood glucose levels, and sustained C-peptide levels. Dapagliflozin stimulated the proliferation of alpha and beta cells while decreasing cell apoptosis. It also facilitated the transdifferentiation from alpha to beta cells, evidenced by an increase in glucagon/PDX-1 positive cells. In vitro studies showed that dapagliflozin had no direct impact on islets, implying that its benefits in vivo are indirect. Overall, SGLT2 inhibition supports the function of human islets and may assist in the regeneration of beta cells in diabetes (Karlsson et al. 2022).

### Prolactin

During pregnancy, the body produces lactogenic hormones, such as prolactin, which can be attached to the prolactin receptor (PLR) (Butler et al. 2010). The PLR then transmits signals downstream through two main pathways: the phosphatidylinositol-4,5-bisphosphate 3-kinase (PI3K)-AKT serine/threonine kinase (AKT) pathway and the Janus kinase (JAK)-signal transducer and activator of transcription (STAT) pathway (Bole-Feysot et al. 1998). Initiation of proliferative factor transcription is triggered when STAT proteins are transported into the nucleus as a result of this signaling cascade. The transcription of two important proliferative factors, osteoprotegerin (OPG) and serotonin (5-HT), is induced by these signaling pathways (Kondegowda et al. 2015; Kim et al. 2010). These molecules are then released into the surrounding cellular environment (islet milieu) to sustain mitogenic signaling. This can occur through an autocrine mechanism, where the cells release these molecules and then respond to them themselves, or through a paracrine mechanism, where neighboring cells respond to the released molecules (Fujinaka et al. 2007; Butler et al. 2010). A study demonstrated that human recombinant prolactin treatment increased in vitro survival of human pancreatic beta cells by 37% but did not lead to an apparent increase in proliferation (Yamamoto et al. 2010).

### Proton Pump Inhibitors (PPIs)

Proton pump inhibitors (PPIs) are frequently prescribed medications aimed at treating acid-related disorders like peptic ulcer disease, gastro-oesophageal reflux disease (GORD), and Zollinger-Ellison syndrome (Czarniak et al. 2022). By blocking H + /K + ATPase, PPIs act as potent suppressors of gastric acid; however, they do elevate gastrin levels because of negative feedback mechanisms. PPIs significantly increase serum gastrin levels, influencing glucose metabolism by promoting beta-cell growth and enhancing insulin secretion. Gastrin is viewed as a promising early incretin candidate, as it stimulates beta cells to secrete insulin in diabetic animal models (Trang et al. 2021). Omeprazole, esomeprazole, lansoprazole, pantoprazole, rabeprazole, and dexlansoprazole are the six PPIs available today. Although they all share a structural similarity, their chemical stability at various pH levels differs (Boj-Carceller 2013).

To address gastritis in diabetic persons, PPIs are sometimes administered alongside metformin or DPP‐4 inhibitors (such as alogliptin, linagliptin, saxagliptin, or sitagliptin) or a combination of the two (Tasnim et al. 2024). In 2014, AnHye Kim and colleagues examined the impact of proton pump inhibitors (PPIs) on the pharmacokinetics and pharmacodynamics of metformin. Their research revealed that the concurrent use of PPIs, specifically pantoprazole and rabeprazole, resulted in a substantial elevation of plasma metformin concentrations. However, the influence of these PPIs on glucose regulation was minimal and varied depending on the specific PPI used (Kim et al. 2014). Also, a study conducted in 2022 by Francisco Alejandro Lagunas-Rangel and colleagues indicated that a triple-drug combination of GABA, sitagliptin, and a PPI (referred to as A + B + C) was more effective in preventing diabetes onset, increasing C-peptide and insulin levels, lowering blood glucose, and reducing the need for external insulin therapy in severely diabetic mice (Lagunas-Rangel et al. 2022).

### Kinase enzymes for beta cell proliferation

#### DYRK1A

DYRK1A is implicated not only in neurodegenerative disorders but also in cellular proliferation. Several investigations reveal that inhibiting DYRK1A promotes cellular growth, increases pancreatic islet mass, and improves glycemic homeostasis in diabetic mice with a deficient quantity of human islets (Shen et al. 2015). In 2012, Annes et al. initially reported a DYRK1A inhibitor that could potentially stimulate beta cell proliferation. The study highlighted that 5-iodotubericidin (5-IT) could induce the replication of rodent and porcine beta cells, an outcome that was initially credited to the inhibitory effects of 5-IT on adenosine kinase (Annes et al. 2012a). DYRK1A inhibitors consist of harmine (Bain et al. 2007), leucettine-41 (Naert et al. 2015), GNF4877 (Liu et al. 2020a), GNF2133 (Liu et al. 2020b), OTS-167 (Allegretti et al. 2020), 5-iodotubercidin (Dirice et al. 2016a), 1,5-naphridine, and CC-401 (Abdolazimi et al. 2018) possess the ability to stimulate the replication of human beta cells (Goode et al. 2022; Jarhad et al. 2018; Cerf 2013; Guo et al. 2022). Most DYRK1A inhibitors function by inhibiting the kinase catalytic activity through competitive inhibition of ATP at its ATP-binding site (Pucelik et al. 2023; Nguyen et al. 2017). Treatment with harmine in an in vitro environment led to an increased proliferation of human pancreatic beta cells, with levels reaching approximately 1.3%. This effect was achieved through the activation of the DYRK1A-NFAT pathway, which has been shown to stimulate the transcription of genes related to cell proliferation, including regulators of the cell cycle and IRS-2 receptors (Wang et al. 2015).

**Fig. 2:**
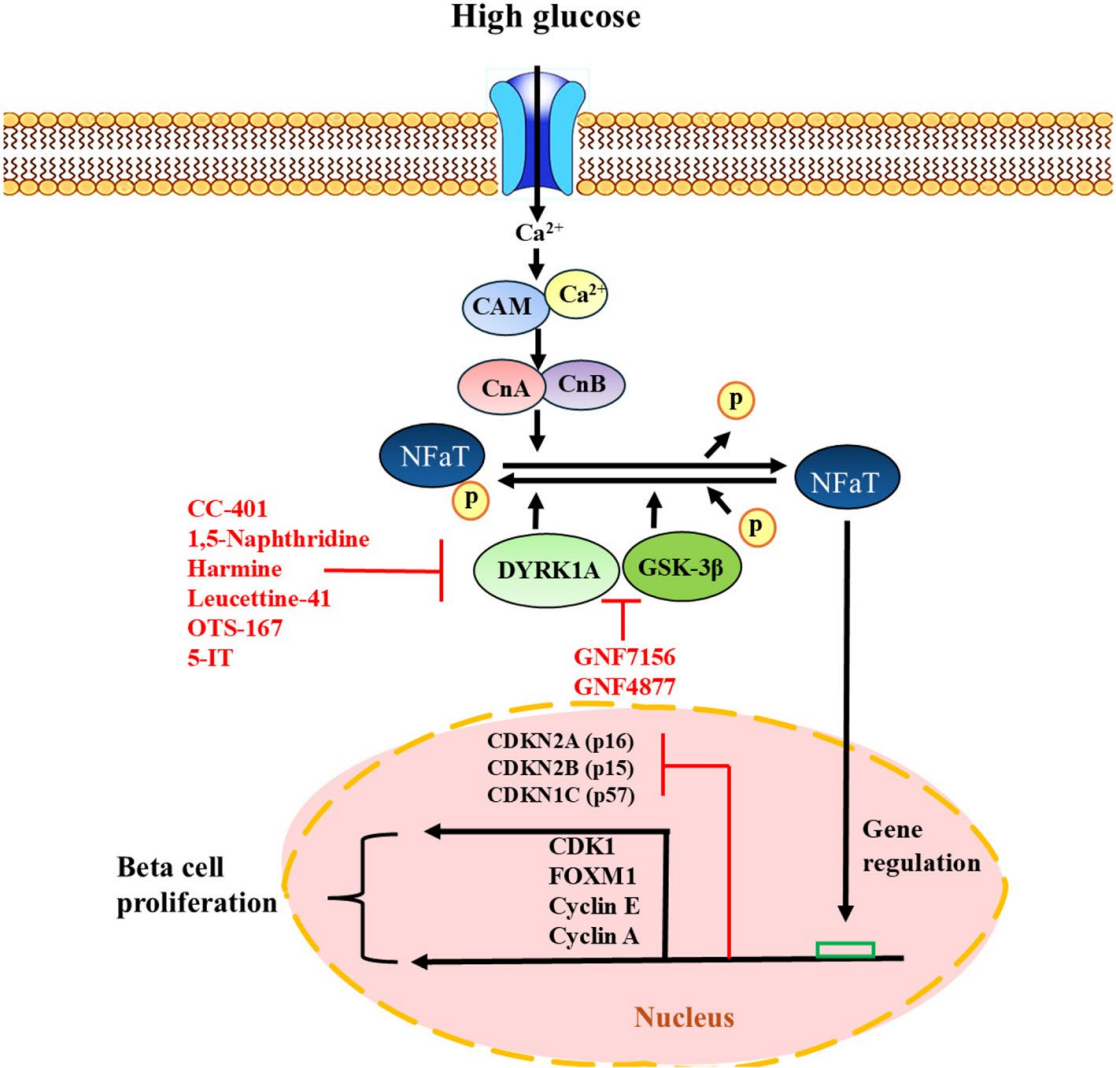
Exposure to elevated glucose levels causes increased intracellular calcium in beta cells and leads to the activation of calcineurin A and B (CnA and CnB). They remove phosphate groups from NFATc proteins, allowing them to enter the nucleus. The CN-NFATc pathway is important for regulating beta cell growth. DYRK1A and Gsk3b kinases add phosphate groups to NFATc proteins, causing them to leave the nucleus. Compounds like GNF4877 inhibit DYRK1A and Gsk3b, keeping NFATc proteins in the nucleus and promoting beta-cell proliferation

By blocking DYRK1A, harmine increases beta cell proliferation in mice and humans by boosting the expression of cell cycle target genes and easing the nuclear translocation of NFAT transcription factors (Fig. 2) (Wang et al. 2015; Kwilas et al. 2015). Numerous combinational trials have been studied to boost the effectiveness of harmine. The simultaneous suppression of DRYK1A and Transforming Growth Factor beta (TGFβ) signaling was demonstrated to effectively enhance the proliferation of human beta cells synergistically while avoiding any detrimental consequences (Wang et al. 2019). Furthermore, the administration of harmine and GLP1 combined with human islets resulted in an average proliferation rate of 5%, comparable to the combination with TGF (Ackeifi et al. 2020b). Moreover, Inhibiting DYRK1A in tandem with inhibiting glycogen synthase kinase-3β18 or the SMAD and trithorax pathways 3, or with activating the glucagon-like peptide 1 receptor, has a deeper effect on proliferation (Wang et al. 2019; Ackeifi et al. 2020; Shen et al. 2015). Although several DYRK1A inhibitors have been implemented for potential therapeutic use in neurological disorders, cancer, and diabetes, developing a DYRK1A inhibitor that performs selectivity towards beta cells while avoiding adverse reactions is a formidable undertaking. As this enzyme is crucially functioning in different cells and pathways, targeting this enzyme in beta cells and specifically within the proliferation-related pathway requires highly DYRK1A-specific antagonists (Allegretti et al. 2020).

#### DYRK1A and GSK-3β inhibitors: GNF7156 and GNF4877

In an experiment, Shen and colleagues used data acquired from previously established high-throughput screening techniques to investigate beta cell proliferation and generate compounds derived from an aminopyrazine scaffold for this purpose. Among the investigated compounds, GNF7156 and GNF4877 were screened as potential candidates for optimal beta cell proliferation (Shen et al. 2015a). The function of these two compounds was verified as a GSK-3β inhibitor. Additionally, it has been demonstrated that these compounds possess the capability to impede the activity of the nuclear factor of activated T-cells (NFAT) kinases. The important point is that they can inhibit DYRK1A in a specific manner (Dirice et al. 2016b). This inhibition ultimately results in the localization of NFAT in the cell nucleus, which is crucial for promoting the proliferation of b-cells (Fig. 2) (Shen et al. 2015a).

#### RANK Inhibitors: Osteoprotegrin and Denosumab

**Fig. 3:**
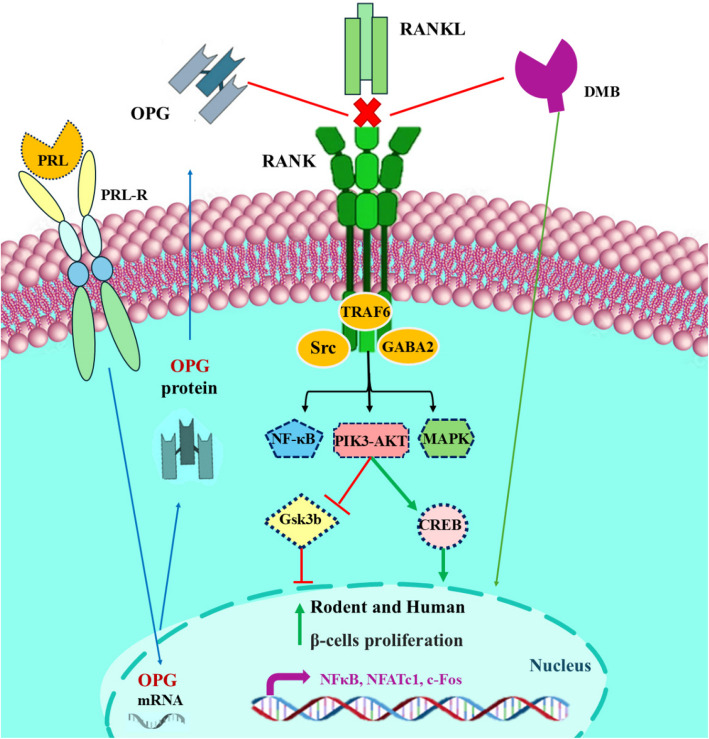
By regulating the CREB and GSK3 pathways, the endogenous inhibitor, osteoprotegerin, and denosumab both suppress the RANKL/RANK interaction and promote the proliferation of b-cells

The findings of Kondegowda and colleagues indicate that osteoprotegerin, a receptor primarily associated with bone-related functions, has the potential to promote the growth of beta cells. In a rodent study, the induction of osteoprotegerin was found to be crucial for lactogen-mediated replication of beta cells. This research indicated that osteoprotegerin can boost the proliferation of beta cells in mice of various ages, including those with diabetes (Vetere et al. 2014). In light of these findings, osteoprotegerin has the potential to be an effective therapeutic target in the management of pancreatic diabetes and beta cell regeneration. Mechanistically, the induction of beta cell replication in both humans and rodents by osteoprotegerin occurs through the modulation of the CREB and GSK3 pathways. This modulation is achieved by binding to the Receptor Activator of NF-κB (RANK) Ligand (RANKL), which acts as a suppressor of beta cell proliferation (Kondegowda et al. 2015). Denosumab (DMB) is a widely used monoclonal antibody for treating osteoporosis and bone metastases of tumors. It basically works by binding to RANKL and inhibiting the interaction with RANK (Fig. 3). Some studies have documented that DMB can stimulate human beta cell proliferation (Vetere et al. 2014; McClung et al. 2006).

#### Protease Inhibitor: SerpinB1

Kulkarni and colleagues adopted a more targeted methodology using a specialized animal model of insulin resistance, liver-specific insulin receptor knockout (LIRKO) mouse, to investigate the crosstalk between the liver and pancreatic islets (El Ouaamari et al. 2016). This study revealed that the liver-derived circulating factors possess the capability to induce the proliferation of pancreatic islets. Among these factors, SerpinB1, also known as leukocyte-neutrophil elastase inhibitor, has been discovered to have the ability to activate the insulin/IGF signaling pathway. This activation leads to the growth of beta cells in zebrafish, mice, and humans. SerpineB1 facilitates the replication of b-cells through the inhibition of serine proteases, such as pancreatic elastase. This subsequently leads to the activation of ERK1 and protein kinase cAMP-dependent type II regulatory subunit b (PRKAR2B) (El Ouaamari et al. 2016; Basile et al. 2022). Consistent with these results, SerpinB1 shows promise as a therapeutic target for cellular diabetes therapy and pancreatic islet regeneration (El Ouaamari et al. 2016; Docherty and Sussel 2021).

#### Glucokinase

Glucokinase is the principal glucose detector in hepatocytes and pancreatic beta cells, and regulating glucose homeostasis is an essential role it performs (Porat et al. 2011). Several reports have documented the effectiveness of small-molecule activators of glucokinase in improving the blood sugar of diabetic animal models. To do this, these activators enhance beta cell insulin secretion and liver glucose uptake (Matschinsky et al. 2011; Vetere et al. 2014). However, In the clinical stage of these activators, some challenges were raised regarding the hypoglycemic effect, heightened liver burden, and diminishing efficacy over time (Thilagavathi et al. 2022). Dorzagliatin is a unique and innovative dual-acting allosteric glucokinase (GK) activator (GKA). It acts by directly binding to a specific pocket located away from the active site of GK, which enhances its ability to bind with glucose (Chow et al. 2023; Zhu et al. 2018). Dorzagliatin is a highly effective medication that has been proven to improve glycemic control in patients with TD2 significantly. Activating glucokinase in a glucose-dependent manner enhances insulin secretion, resulting in better management of blood sugar levels. This medication is administered orally and is quickly absorbed by the body, making it a convenient and reliable option for diabetic patients (Zhu et al. 2022). Several trials have been undertaken utilizing the hepatoselective activator TTP399, targeting enhanced glycemic regulation in TD1 patients, whereas the majority of glucokinase activator trials have focused on TD2 in humans (Goode et al. 2022; Klein et al. 2021). TTP399 has demonstrated remarkable potential in reducing glycated hemoglobin levels in a clinically significant and sustained manner as a selective activator of GK. This hypoglycemic effect and further stimulation of beta cell proliferation pose minimal risk of adverse effects, making it a hopeful option for the fundamental and capable therapy of both types of diabetes (Klein et al. 2022).

#### Adenosine kinase

A study by Annes JP et al. presents the progress of a screening platform focused on the discovery of small-molecule compounds that can enhance the replication of beta cells. Through the utilization of this platform, they were able to successfully identify a specific category of compounds known as adenosine kinase inhibitors (ADK-Is), that have shown the capacity to induce the replication of primary beta cells across multiple species, including mice, rats, and pigs (Annes et al. 2012b). It is noteworthy that the replication-inducing effect of ADK-Is exhibits a certain level of selectivity towards specific cell types. The administration of ADK-Is to islet cell cultures has been noted to enhance beta cell replication exclusively, without any significant influence on the replication of α-cells, PP cells, or fibroblasts (Annes et al. 2012b). In the experiment of rat islets, two inhibitors of adenosine kinase, namely 5-iodotubercidin (5-IT) and ABT-702, were discovered as potential ones. These inhibitors were found to exert their effects on the proliferation of beta cells through the action of a nuclear isoform of ADK and, in a manner, dependent on mTOR (Annes et al. 2012b; Vetere et al. 2014; Goode et al. 2022). Furthermore, the adenosine agonist 5′-N-Ethylcarboxamidoadenosine (NECA) emerged as the most potent stimulator of beta-cell regeneration in zebrafish. The mechanism of action involves the activation of the adenosine receptor, ADORA2A, resulting in enhanced beta cell proliferation and expedited restoration of normoglycemia (Andersson et al. 2012).

#### IκB Kinase and EBP1 Inhibitors

Shen et al. have performed a high-throughput assessment of ~ 850,000 heterocycles in a cell-based assay, utilizing rodent beta cell lines R7T1 to find compounds that promote the proliferation of beta cells (Shen et al. 2013). Through their screen, a diarylurea WS1 was discovered, which is a chemical compound capable of initiating cellular proliferation. Following this, they proceeded to synthesize an analogous compound, diaryl amide WS6, which exhibited the ability to enhance the proliferation of R7T1 cells (Shen et al. 2013). The affinity purification method and tandem mass spectrometry found that WS6 can bind and block ERBB3 binding protein 1 (EBP1) and inhibitor of NF-κB kinase subunit ε (IKKε). EBP1 is a well-known suppressor of the cell cycle. When interacting with histone deacetylases and retinoblastoma protein, the suppression of EBP1 can release the cell cycle and lead to further proliferation. IKKε is involved in regulating the nuclear factor κB (NF κB) pathway (Kraan et al. 2014). These findings bring the idea together that EBP1 inhibitors can be a practical option for beta cell regeneration in the case of pancreas degeneration.

#### Salt-inducible kinases

The members of the Salt-inducible kinases (SIK) family, consisting of SIK1-SIK3, are recognized as serine/threonine kinases that belong to the AMP-activated protein kinase (AMPK) family (Sun et al. 2020)***.*** SIK2 has demonstrated an essential role in beta cell function, as the glucose-intolerant phenotype arises in beta cells–specific Sik2 knockout animals due to compromised insulin secretion (M. Kim 2004). In a recent work by Charbord et al., a novel method using a luminescence ubiquitination-based cell cycle indicator (LUCCI) in zebrafish was employed to discover HG-9–91-01 as a driver for cellular proliferation, which was subsequently verified in both mouse and human beta cells (Wagner 2022; Charbord et al. 2021). Mechanistic investigations propose that the induction of beta cell proliferation by HG occurs through the integration of multiple signaling pathways. These pathways include ATF6-IRE1, CRTC1-CRTC2, and mTOR, all of which have been individually recognized for their ability to stimulate beta cell proliferation (Hussain et al. 2006; Xu et al. 2014; Blandino-Rosano et al. 2012). In a separate investigation carried out by Iorio et al., the enhancement of human beta cells was demonstrated to be triggered by selectively focusing attention on the GPCR GPR3-SIK2 pathway. Deprivation of GPR3 results in the activation of Salt salt-inducible kinase 2, a crucial and definitive factor in promoting the initiation of the cellular cycle, enhancing the mass of beta cells, and increasing insulin secretion in mice (Iorio et al. 2021). Surprisingly, the research conducted by Iorio et al. demonstrated that the pan-SIK inhibitor HG-9–91-01, when applied at a concentration of 2 μM, effectively hindered the process of cell proliferation induced by GPR3 silencing. Conversely, in the investigation conducted by Charbord et al., the same inhibitor, at concentrations ranging from 0.1 to 1 μM, was observed to stimulate proliferation in zebrafish, mice, and human beta cells. Notably, the impact on proliferation was negligible when HG-9–91-01 was administered at a concentration of 3 μM (Goode et al. 2022; Charbord et al. 2021; Iorio et al. 2021). Furthermore, recent evidence has shown that HG-9–91-01 can effectively inhibit the activity of the kinase RIPK3 within comparable dosage intervals (0.5~5 μM). RIPK3 is essential in modulating TNFα-induced beta cell death in TD1 mouse models (Huang et al. 2022; Contreras et al. 2022).

## Signaling pathways containing drug-induced targets in beta cell proliferation

In the treatment of diabetes, there are various potential therapeutic targets that regulate β‑cell proliferation through different cellular signaling pathways (Table 3).

1. Insulin signaling pathways: PI3K-AKT/PKB signaling pathway, calcium-mediated signaling pathway, RAS-extracellular signal-regulated kinase (ERK)/MAPK signaling pathway (Stewart et al. 2015).
2. Growth factor signaling pathways: EGF family, NGF, TGFβ superfamily, PLGF, and Pref-1 signaling pathway (Heydarpour et al. 2020).
3. Hormone signaling pathways: Hormones such as thyroid hormone (TH), growth hormone (GH), glucagon-like peptide 1 (GLP-1), IGF-1, and prolactin (PRL) as well as their corresponding receptors (TR, GHR, GLP-1R, IGF-1R, and PRLR, respectively) have been significantly linked to the survival, growth, proliferation, differentiation, and insulin secretion of beta cells. These hormones may also be involved in the interaction between PI3K-AKT, JAK-STAT, and ERK (Huang and Chang 2014).
4. Wnt signaling pathways: The wnt signaling pathway typically consists of extracellular Wnt ligands, disheveled protein, Frizzled receptor, β-catenin, axin, GSK3β, and adenomatous polyposis colitis protein (Xue et al. 2016).
5. JAK-STAT signaling pathways: The JAK-STAT pathway has been linked to various cytokines, including interleukin (IL)−1, −2, and −6, as well as hormones like leptin, GH, placental lactogens, erythropoietin (EPO), and PRL (Stewart et al. 2015).
6. TLR4 signaling pathway: A gene potentially implicated in the onset of DM is toll-like receptor 4 (TLR4), predominantly found in innate immune mechanisms (Shao et al. 2014).

**Table 3:** Signaling pathways, therapeutic targets and mechanistic effects in trials of induced beta cell proliferation

Signaling pathway	Target	pharmacological agents	Effect of agent at Target	Mechanism	Measuring methods	Ref
Insulin signaling pathways	DYRK1A	Harmine 5-IT GNF4877	Inhibition	Reduces NFAT phosphorylation	In-vitro In-vitro, In-vivo In-vitro, In-vivo	Bain et al. 2007; Dirice et al. 2016a; Liu et al. 2020a
	CREB	Osteoprotegerin	Activation	Inhibit RANKL from binding to RANK	In-vivo	Kondegowda et al. 2015
	Neutrophil elastase (NE)	SerpinB1	Inhibition	Increasing the phosphorylation levels of MAPK	In-vivo	El Ouaamari et al. 2016
	Gaba receptor	Gaba	Activation	GABA stimulates beta cell replication in transplanted human islets	In-vivo	Purwana et al. 2014b; Hagan et al. 2022; Soltani et al. 2011
Growth factor signaling pathways	TGF-βR	SB431542	Inhibition	Increases cell proliferation by blocking SMAD3 activation	In-vitro	Lin et al. 2009
Hormone signaling pathways	IR/IGF1R	Insulin IGF1	Activation	Trigger beta cell regeneration by regulating the PI3K-AKT axis	In-vitro	Hü et al. 1998
	GLP1-R	GLP-1 Exendin-4	Activation	Trigger beta cell regeneration by regulating the PI3K-AKT axis	In-vitro, In-vivo	Fusco et al. 2017
Wnt signaling pathways	GSK3β	GNF7156 GNF4877	Inhibition	Unknown	In-vitro, In-vivo	Liu et al. 2020a; Shen et al. 2015
	GSK3β	Osteoprotegrin Denosumab	Inactivation	Prevent the interaction of RANKL with RANK	In-vitro, In-vivo	Kondegowda et al. 2015; McClung et al. 2006
	GSK3β	SerpinB1	Inhibition	Increasing phosphorylation levels of GSK3β	In-vivo	El Ouaamari et al. 2016
JAK-STAT signaling pathways	Prolactin receptor (PLR)	Recombination human Prolactin (rhPRL)	Activation	Adding rhPRL to islet culture media enhanced human beta cell survival without compromising islet quality	In-vitro	Yamamoto et al. 2010
TLR4 signaling pathway	NFκB	WS6	Inhibition	Enabling NFκB to move into the nucleus and stimulate cell growth	In-vitro	Shen et al. 2013

Due to the variable involved in signaling pathways and potential targets, there are several potential approaches for further investigations and the development of therapeutic targets to address diabetes.

## Clinical Challenges

### Translational barriers

Preclinical studies in rodents and other animal models have demonstrated encouraging results in drug-induced regeneration of pancreatic beta cells. However, applying these findings to human clinical settings creates considerable challenges due to the complexity of human physiology and interspecies differences, which pose concerns about efficacy, safety, and scalability (Dirnagl et al. 2022). Ensuring the safety and long-term effectiveness of regenerative therapies is crucial. Although preclinical studies are promising, applying these findings to humans has proven challenging. Many regenerative molecules that work in animal models fail during human trials. Off-target effects can pose risks, such as tumor development or immune responses. Long-term studies are necessary to assess the ongoing benefits and risks of these therapies, ensuring that beta-cell regeneration does not lead to new health problems (Wang et al. 2021; Levetan and Pierce 2013). The replicative capacity of human and rodent B cells shares both similarities and differences. For instance, in both species, B cell mass grows during early life stages and decreases as they age. However, during pregnancy and obesity, the adaptive b cell proliferation occurs extensively in rodent beta cells, but is limited in humans (Levetan and Pierce 2013). During pregnancy, insulin resistance leads to increased insulin production to keep glucose levels stable. In rodents, this rise in insulin production is linked to an increase in the number of beta cells, which is influenced by lactotrophic hormones (Xu et al. 2015). However, in humans, the proliferation of beta cells stimulated by lactotrophic hormones or other growth-inducing factors is limited (Baeyens et al. 2016). Mitogenic agents, hormones, and growth factors (GFs) like Glp-1, Gip-1, exendin-4, prolactin, Hgf, and Igf-1 are known to stimulate beta cell proliferation in rodent models. These substances play a crucial role in the regulation of cell growth and division, contributing to the maintenance and expansion of beta cell populations in these animals. However, despite their effectiveness in rodent B cells, these agents do not induce the same proliferative response in human beta cells. This discrepancy highlights the species-specific differences in cellular signaling pathways and the challenges in translating findings from animal models to human biology. Understanding these differences is essential for developing effective therapies targeting beta cell regeneration in humans (Tella and Rendell 2015; Fusco et al. 2017; Menge et al. 2008; Saisho et al. 2013).

### The Promise and Pitfalls of DYRK1A Inhibitors

DYRK1A inhibitors show significant promise in preclinical models by promoting human beta cell proliferation and enhancing beta cell function. However, several critical limitations have hindered their advancement to clinical trials. A primary concern is the lack of selectivity among current DYRK1A inhibitors (Chen et al. 2024). No small molecule inhibitor targeting DYRK1A is entirely selective; all tested inhibitors also affect numerous other kinases, leading to worries about off-target effects and potential toxicity in humans (Ullsten et al. 2025). For instance, GNF4877 interacts with 254 out of 468 human kinases, indicating high promiscuity (Shen et al. 2015c). Similarly, 5-iodo-tubericidin (5-IT) inhibits 102 kinases. Even relatively selective inhibitors like harmine and compound 2-2c inhibit 8~9 kinases, including DYRK1A, DYRK1B, DYRK2, and members of the CLK family (CLK1, CLK2)(Dirice et al. 2016c; Kumar et al. 2020). The broad inhibition raises significant concerns about off-target effects that could lead to toxicity or unintended physiological outcomes if applied in humans. For example, while GNF4877 effectively inhibits DYRK1A, its lack of selectivity likely disrupts pathways essential for beta cell differentiation, as it was found to block harmine's ability to promote differentiation. This indicates that some inhibitors might even counteract desired therapeutic effects due to their off-target activities (Dirice et al. 2016c). The mechanism by which DYRK1A inhibitors enhance beta cell differentiation remains unclear. Initially, it was believed that inhibiting DYRK1A would promote both proliferation and differentiation, but experiments showed that silencing DYRK1A induced proliferation without affecting differentiation markers (Wang et al. 2024). This unexpected result suggests that compounds like harmine, 2-2c, and 5-IT might target other unknown entities beyond DYRK1A. Additionally, while harmine, 2-2c, and 5-IT improve differentiation and function, most other DYRK1A inhibitors do not, highlighting inconsistency within this class. Efforts to pinpoint other responsible targets by examining related kinases like DYRK1B, DYRK2, DYRK3, DYRK4, the CLK family, the DREAM complex, and NFAT proteins were unsuccessful, as silencing these did not replicate the differentiation effects (Ackeifi et al. 2020). The exact “pro-differentiation target” is still unclear, likely needing unbiased proteomic approaches for identification. Challenges like low selectivity, potential off-target effects, and incomplete mechanism insights explain why DYRK1A inhibitors, despite encouraging in vitro and animal model outcomes, have not advanced to clinical trials for diabetes therapy (Wang et al. 2024).

## Conclusions and perspectives

Among the most basic ways to treat DM, drug-induced regeneration of pancreatic beta cells shows promise. However, there is significant knowledge of studies that highlighted the critical biological aspects of regeneration, exploring the mechanisms and cellular activities that enable the regeneration and renewal of beta cells (Bourgeois et al. 2024); in this study, we shift the attention to the pharmacological agents used in beta cell regeneration trials and their intracellular targets and beneficial points achieved in the previous trials. These data can explain the ways in which medications and therapeutic agents may enhance the regeneration of insulin-secreting beta cells in the pancreas practically for the field researchers. By exploring pharmacological strategies that promote beta cell growth and function, we can select the optimal and improved targeted treatments in the future diabetes regeneration trials of diabetes in clinical regenerative medicine. This review has indicated the importance of targeting specific enzymes involved in the regeneration process as potential therapeutic targets. By modulating the activity of these enzymes, it is possible to enhance the regeneration of pancreatic beta cells and improve their function. Several key enzymes have the potential to be pharmacologically induced for optimal beta cell regeneration. These include enzymes involved in cell proliferation, differentiation, and survival pathways. Targeting these enzymes with specific drugs or small molecules holds great potential for promoting beta-cell regeneration and restoring normal glucose homeostasis in individuals with diabetes. Current agents are in their early stages, focusing on promoting beta cell differentiation. However, directly altering transcription factors is complex, and more upstream biomolecular targets are being sought. One significant discovery in this field is the identification of DYRK1A, a key protein kinase that plays a role in regulating beta cells. This discovery offers possibilities for diabetes treatment, as DYRK1A operates in the proliferation and differentiation of beta cells, presenting it as an appropriate target for enhancing beta cell regeneration. Modulating the activity of DYRK1A kinase toward beta cell differentiation suggests that the “diabetic kinome” could be a goal for future diabetes therapies. Both scientific research and the pharmaceutical industry have recognized the significance of DYRK1A kinase in various molecular processes, indicating its potential as a target for developing new treatments for diabetes. Nonetheless, there have been efforts to develop and study DYRK1A inhibitors for the potential treatment of various conditions, including neurological disorders, cancer, and diabetes. However, the challenge lies in finding an inhibitor that specifically targets beta cells without causing adverse reactions. One potential solution is to use putative beta cells targeting ligands to deliver the inhibitors, ensuring they are exclusively delivered to the beta cells. In addition to this, there are other suggested methods for transferring inhibitors of this enzyme to beta cells in a specific manner, including Antibody–Drug Conjugates, Peptide-Drug Conjugates, Small Molecule-Drug Conjugates, and Zinc Chelating Small Molecule-Drug Conjugates. Given the significance of this enzyme in beta cell growth and proliferation, these strategies seek to guarantee the specificity of compounds that inhibit it. Therefore, choosing this enzyme for future studies is a promising option and demands a sophisticated screening of inhibitors within in vitro and in vivo trials of pancreatic regeneration.

## Data Availability

Not applicable.

## Abbreviations

DM: Diabetes mellitus
TD1: Type 1 diabetes
TD2: Type 2 diabetes
DYRK1A: Dual Specificity Tyrosine Phosphorylation-Regulated Kinase 1A
SIK: Salt-Inducible Kinase
TGF-β: Transforming Growth Factor-β
EGF: Endothelial Growth Factor
GLP1: Glucagon-Like Peptide-1
CDK: Cyclin-Dependent Kinase
GABA: Γ-Amino Butyric Acid
GLP1R: Glucagon-Like Peptide 1 Receptor
GPCR: G Protein–Coupled Receptor
hESC: Human Embryonic Stem Cells
hiPSC: Human Induced Pluripotent Stem Cell
IL: Interleukin
NFAT: Nuclear Factor of Activated T Cells
TGF: Transforming Growth Factor
TNF: Tumor Necrosis Factor
DPP-4: Dipeptidyl Peptidase-4
PPIs: Proton Pump Inhibitors
